# Vestibular Evoked Myogenic Potentials Are Abnormal in Idiopathic REM Sleep Behavior Disorder

**DOI:** 10.3389/fneur.2018.00911

**Published:** 2018-10-29

**Authors:** Edoardo Rosario de Natale, Francesca Ginatempo, Ilaria Laccu, Michela Figorilli, Andrea Manca, Beniamina Mercante, Monica Puligheddu, Franca Deriu

**Affiliations:** ^1^Department of Biomedical Sciences, University of Sassari, Sassari, Italy; ^2^Department of Medical Sciences and Public Health, Sleep Disorder Research Center, University of Cagliari, Cagliari, Italy

**Keywords:** vestibular evoked myogenic potentials, REM sleep behavior disorder, brainstem, neurodegeneration, neurophysiology

## Abstract

**Objectives:** To investigate brainstem function in idiopathic REM sleep Behavior Disorder (iRBD), a condition occurring as a result of a derangement of connections within brainstem structures, with a battery of Vestibular Evoked Myogenic Potentials (VEMPs), neurophysiological tools suited for the functional investigation of the brainstem. Neurophysiological data were correlated with clinical characteristics of patients.

**Methods:** Twenty patients with iRBD and 22 healthy controls underwent cervical (cVEMP), masseter (mVEMP) and ocular (oVEMP) VEMP recording. Patients were assessed clinically according to presence of motor as well as non-motor symptoms such as constipation, depression, and hyposmia. Also, they were screened for postural instability through the Berg Balance Scale (BBS). VEMPs were categorized as for increasing degrees of abnormalities, namely latency delay, amplitude reduction and absence; a VEMP score was built accordingly.

**Results:** Compared with controls, iRBD had higher rates of abnormalities both in the VEMP battery (iRBD 75%, Controls 23%; *p* < 0.01) as well as in each single VEMP (cVEMP: 45 *vs*. 5%; mVEMP: 65 vs. 13.6%; oVEMP: 50 vs. 5%; *p* < 0.01), which exhibited significantly lower amplitudes (cVEMP and oVEMP: *p* < 0.0001; mVEMP: *p* = 0.001) in iRBD. Within altered reflexes, absence was predominant in oVEMP (81%), amplitude reduction in mVEMP (50%) and cVEMP (70%). Severity of VEMP alterations was significantly higher in iRBD compared with controls (*p* < 0.05 for all VEMPs), as indicated by the larger VEMP scores in the former. The oVEMP score correlated inversely with poor performances on the BBS.

**Conclusion:** VEMPs unveil consistent and extensive brainstem abnormalities in iRBD patients. Further studies are warranted for testing the potential of VEMPs in the monitoring of the evolution of iRBD over time.

## Introduction

REM Sleep Behavior Disorder (RBD) is a disease in which patients lose their normal muscle paralysis during REM sleep and actively enact their dreams (1). This occurs as a result of a breakdown of complex connections between regions that mediate sleep atonia in the brainstem involving monoaminergic (such as in the locus coeruleus/subcoeruleus complex and the median raphe) and non-monoaminergic (such as the pedunculo-pontine region) neurotransmitters (2). It has been established that, in a high percentage of cases, RBD represents the early manifestation of a number of neurodegenerative diseases, most frequently synucleinopathies (3) such as Parkinson's Disease (PD), in which the initial target of the neuropathological process is constituted by a brainstem degeneration, according to a proposed caudo-rostral progression (4). The mechanisms of how neurodegeneration occurs and progresses are, however, still unclear and active research is underway in the quest for reliable clinical biomarkers that would be helpful for identifying and monitoring disease progression.

In PD, the functional integrity of the brainstem has been recently investigated through a battery of vestibular-evoked myogenic potentials (VEMPs), composed of the cervical (cVEMP), the masseter (mVEMP) and the ocular (oVEMP) VEMPs. These three reflexes are able to provide useful information if brainstem integrity along its whole length, from the cervico-bulbar junction to the upper brainstem. In previous studies, a significant rate of VEMPs alterations since the earliest motor stages of the disease were reported as well as a strong association between VEMPs abnormalities and the presence of symptoms suggestive of RBD as assessed through the RBD-SQ (5, 6). Furthermore, VEMPs have been used to detect functional alterations of the brainstem that can be hardly identified with other means of investigation (7, 8). In RBD the available literature on neurophysiological tests of the brainstem is scarce: is it limited to single case reports (9) or to cross-sectional studies employing the Prepulse Inhibition (PPI) in the differentiation of iRBD from PD or Multiple System Atrophy patients (10).

In this light, we hypothesized that a study of the brainstem in RBD through a battery of VEMPs would provide more complete information about a possible brainstem disruption. Therefore, aims of this study were to investigate the brainstem function in a cohort of idiopathic RBD patients through a battery of VEMPs and to correlate VEMPs alterations with clinical data and presence of other symptoms/signs in co-morbidity which might be warning functional signs of neurodegeneration.

## Methods

### Participants

A total of 42 subjects were recruited for the study: 20 patients with polysomnographic-proven idiopathic RBD according to AASM criteria (https://aasm.org/resources/pdf/scoring-manual-update-april-2017.pdf) and 22 age- and gender-matched healthy controls. All patients were recruited from the Center of Sleep Disorders of the University of Cagliari. For both patients and controls, the following exclusion criteria were considered: presence of cervical orthopedic and stomatognathic diseases; history of peripheral ear/vestibular disorders or inadequate auditory acuity; cerebello-pontine disorders and migraine; postural instability and vertigo from known causes; use of anti-epileptic drugs, benzodiazepines and anti-depressant drugs in the 3 months preceding the study, and a score >0 on the Unified Parkinson's Disease Rating Scale, part 3 (UPDRS-III). A preliminary neuro-otologic evaluation was conducted in all participants to exclude any central and/or audio-vestibular disorder. Additionally, patients affected by other sleep disorders, primarily sleep apnea and periodic limb movements, were excluded. The protocol of this study was approved by the Ethic Committee of Sassari ASL (prot. 987/2 of 24/11/2015). All participants signed an informed consent in accordance with the Declaration of Helsinki on studies involving human subjects.

### Clinical assessment

Clinical screening assessment consisted in motor examination through the administration of the UPDRS scale part III. iRBD patients were excluded if they had scored more than 0 on this scale. A recording of the presence of non-motor symptoms (hyposmia, autonomic dysfunction, depression/anxiety) that may be associated with iRBD for future development of neurodegenerative disorders was taken through anamnestic history or medical records. Furthermore, a screening for presence of postural instability through administration of the Berg Balance Scale (BBS) (11) was performed.

### Polysomnographic recording

All patients underwent one full-night attended video-polysomnography (video-PSG) recording in a sleep laboratory with digital polysomnography according to the American Academy of Sleep Medicine (AASM) recommendations. Video-PSG was performed with digitally synchronized videography and the following montage was employed: electroencephalographic leads (F3-A2, F4-A1, C3-A2, C4-A1, O1-A2, O2-A1), left and right electrooculography (EOG) channels, bilateral surface EMG channels (submentalis, flexor digitorum superficialis on upper limbs, tibialis anterior on lower limbs), and electrocardiography. The respiratory analysis included nasal airflow, which was recorded by both thermistor and nasal pressure sensor, thoracic and abdominal respiratory effort, oxygen saturation recording by cutaneous finger pulse-oxymeter and microphone. Patients were asked to sleep uncovered to improve the detection of motor activity, but a light sheet could be allowed for their comfort.

Sleep stages were scored according to AASM criteria, with allowance to chin EMG muscle tone during REM sleep.

### Neurophysiological evaluation

#### VEMPs recording

Both iRBD patients and controls underwent recording of cVEMP, mVEMP and oVEMP. All participants were seated in a dim and quiet room with support for head, neck and arms. Subjects were instructed to contract steadily the target muscles with the aid of visual feedback during the recordings. For cVEMPs recording, the sternocleidomastoid muscles were contracted by pushing the forehead against the examiner's hand; for mVEMP, activation of the masseter muscles was achieved by occluding the teeth at 30–50% of their maximal voluntary contraction; finally, to record the oVEMP, the inferior oblique muscles were activated by staring at a target about 30° over the eyes. For all VEMPs, vestibular stimulation was produced by loud clicks (300–500 stimuli of 0.1 ms, frequency 5 Hz, intensity 138 dB SPL) generated by an attenuator (3505 HP attenuator, Cambridge Electronic Design LTD, Cambridge, UK) driven by a software (Signal 5.0 script for VEMP, Cambridge Electronic Design LTD, Cambridge, UK). Click stimulation was delivered monoaurally or binaurally through calibrated stereophonic earphones (TDH-49P earphones, Telephonics, Huntington, NY), during voluntary contraction of the target muscles. Rectified and unrectified EMG activity was recorded bilaterally (1902 quad system amplifier, Cambridge Electronic Design LTD, Cambridge, UK), amplified (x3000), filtered (5–5,000 Hz) and sampled (10 kHz) within a temporal window of 200 ms (50 ms pre-stimulus and 150 ms post-stimulus), using an analog/digital converter (1401 power, Cambridge Electronic Design LTD, Cambridge, UK) with an acquisition and analysis software (Signal 5.0) on PC. EMG signal was recorded using 9-mm-diameter Ag-AgCl surface cup electrodes placed over the target muscles in a belly-to-tendon montage as follows: for cVEMP, active electrodes were positioned on the upper portion of SCM, 8–12 cm from the sternal insertion, reference electrodes on the sterno-costal junction over the sternum and ground electrode at the center of the sternal manubrium (12); for mVEMP, active electrodes were positioned over the belly of the masseter, 2 cm above the mandible angle, reference electrodes on the mandible angle and the ground electrode on the forehead (13, 14); finally, for oVEMP, active electrodes were positioned 1 cm below the inferior eyelids, reference electrodes about 15 mm below the active ones and ground electrode on the forehead (15).

#### VEMPs analysis

For each VEMP, the unrectified EMG was used to measure onset and peak latencies of the first wave (i.e., p13 for the cVEMP, p11 for the mVEMP and n10 for the oVEMP) and peak latency of the second wave (i.e., n23 for the cVEMP and p15 for the oVEMP). The second wave of the mVEMP (n15) was not evaluated as it is undetectable in normal hearing people (16, 17), due to its overlapping with a p16/n21 wave of cochlear origin. Thus, in normal-hearing people, the click induced potential in masseter muscles appears as a p11/n21 complex. The inter-side latency difference between the peaks was also measured. Wave amplitudes (p13/n23 for cVEMP, p11 for mVEMP and n10/p15 for oVEMP) were expressed as a ratio with background EMG activity. The resulting corrected amplitudes were normalized, for comparisons with controls, by converting them into their logarithms (18). Amplitude asymmetry ratio was calculated as reported by Rosengren et al. (19). Criteria for abnormality were set, for both patients and controls, regardless of the stimulation/recording side, as follows: (i) peak latencies above 2.5 SD of control values; (ii) normalized corrected amplitudes below 2.5 SD of control values; (iii) absence of the response. According to previous studies (5, 6), to estimate severity of impairment detected in each VEMP recording of every subject, a VEMP score was implemented according to the degree of the VEMP alteration found in every recording, following previous studies (20–22). A normal VEMP recording was labeled as 0, a delay in the latency of the p1/n1 wave as 1, a reduction in amplitude as 2, and absence as 3. Mean VEMP scores ± SD were calculated for each single VEMP as well as for the whole VEMP battery (total VEMP score).

### Statistical analysis

Statistical analysis was performed with SPSS 20.0 for Windows (Chicago, IL). Comparison between groups was performed with Student's *t*-test for parametric variables and Mann-Whitney *U*-test for non-parametric variables. Comparison between frequencies was calculated with χ^2^ test. Correlations were performed by using Spearman's correlation coefficient. Significance was set as *p* < 0.05.

## Results

The two groups of participants were not significantly different as for age and gender. With regards to the iRBD cohort, signs of depression and constipation were found in 20% and hyposmia in 45% of patients. All iRBD patients scored 0 on the UPDRS part III scale. All demographic and clinical characteristics of the subjects enrolled in the study are summarized in Table 1.

**Table 1:** Demographic and clinical characteristics of the iRBD and control subjects.

	**iRBD (*n* = 20)**	**Controls (*n* = 22)**	***p*-value**
Mean age ± SD (years)	68.75 ± 1.49	66.55 ± 10.19	0.07
Gender (M: F)	16: 4	14: 8	0.31
BMI	27.8 ± 4.27	–	–
Disease Duration (years)	4.75 ± 2.7	–	–
BBS score (/56)	54.3 ± 3.4	–	–
Constipation	4/20	–	–
Depression	4/20	–	–
Hyposmia	9/20	–	–

*iRBD, idiopathic REM Sleep Behavior Disorder; SD, Standard Deviation; BMI, Body Mass Index; BBS, Berg Balance Scale. Statistics: student's t-test and χ^2^ test*.

**Figure 1:**
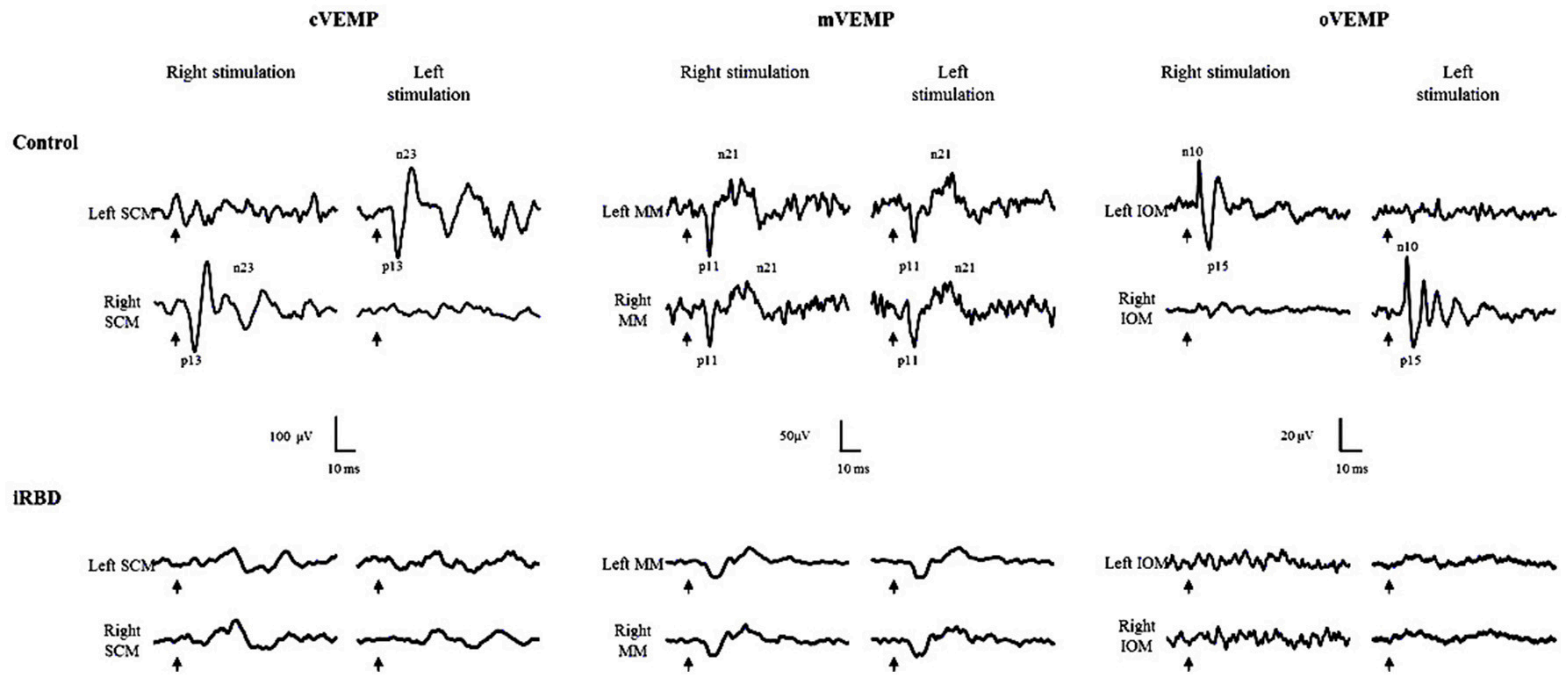
Cervical (cVEMP), masseter (mVEMP) and ocular (oVEMP) VEMPs from sternocleidomastoid (SCM), masseter (MM) and inferior oblique (IOM) muscles in controls and RBD. In patients, oVEMPs and cVEMPs are absent and amplitude of mVEMP is reduced. Arrows: time of stimulus delivery.

Figure 1 reports a set of VEMPs recorded from representative control and iRBD subjects.

**Figure 2:**
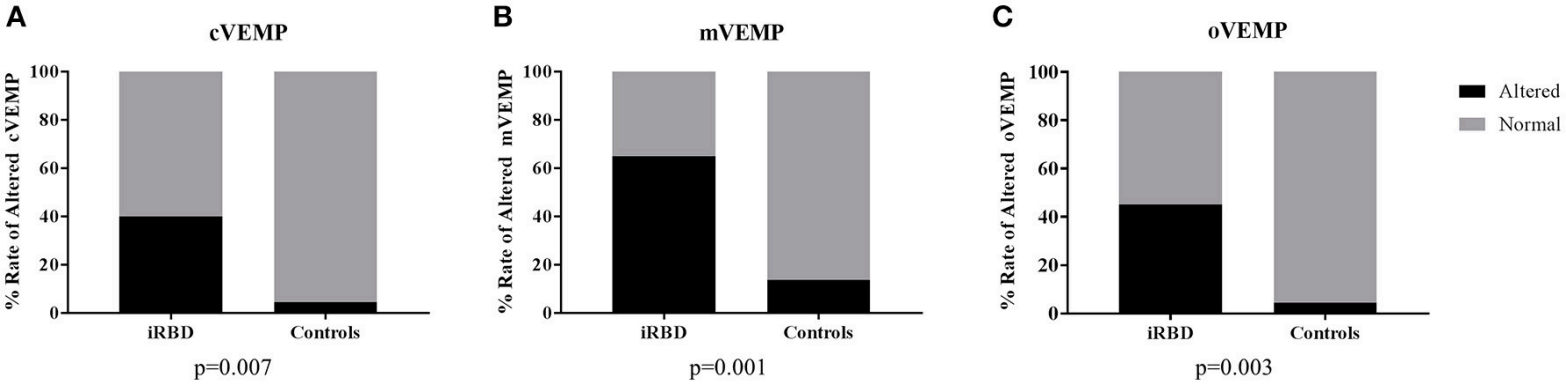
Rates of alterations of each VEMPs in patients and controls. **(A)** cVEMP, cervical VEMP; **(B)** mVEMP, masseter VEMP; **(C)** oVEMP, ocular VEMP; iRBD, idiopathic REM Behavior Disorder. Statistics: χ^2^ test.

**Figure 3:**
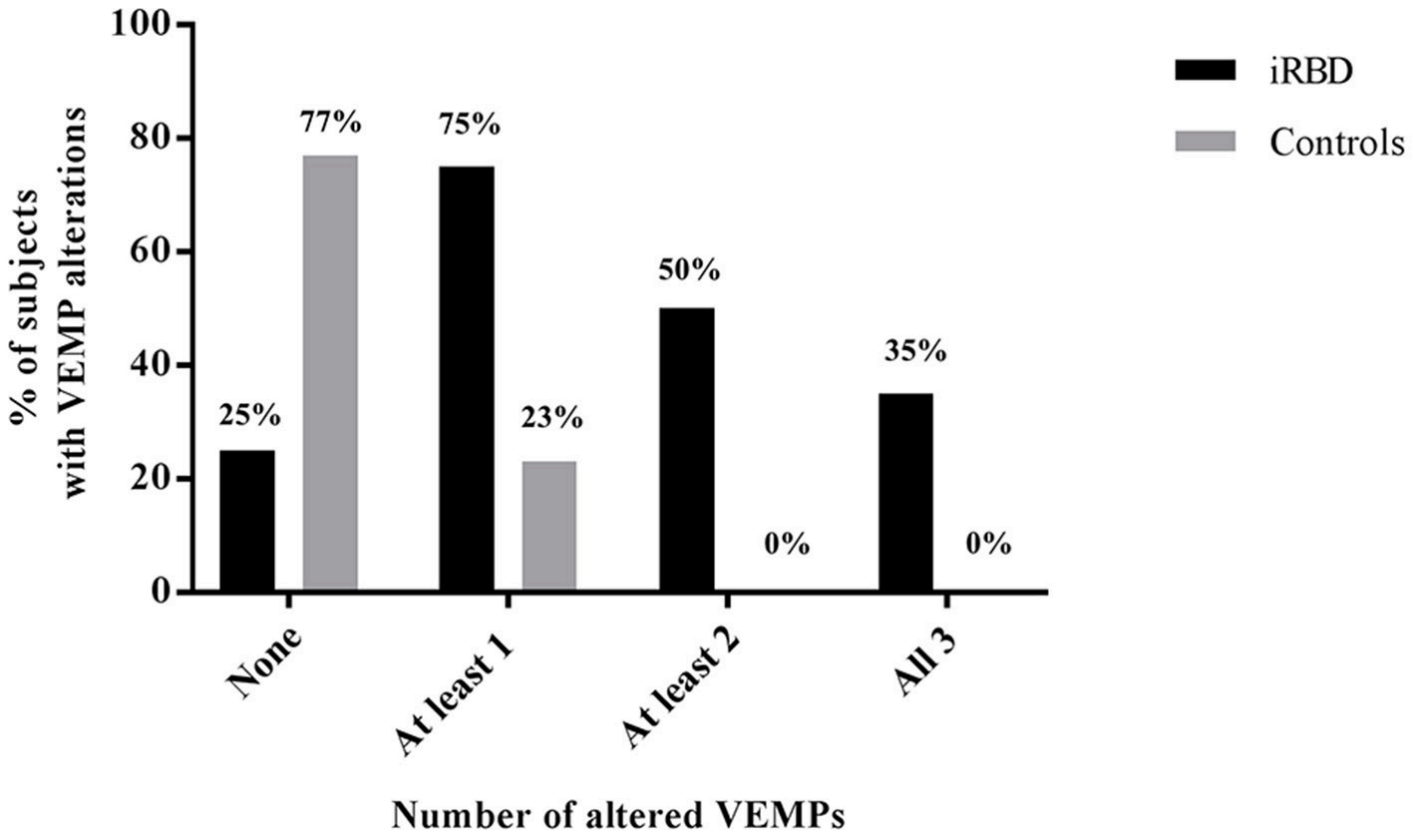
Cumulative percentage of patients and controls subjects displaying alterations in none, one, two, or all the three VEMPs.

For each VEMP tested, there was a consistently higher rate of alterations in iRBD compared with controls (cVEMP: 45 vs. 4.5%, *p* = 0.007; mVEMP: 65 vs. 13.6%, *p* = 0.001; oVEMP: 50 vs. 4.5%, *p* = 0.003) (Figure 2), with only 5 out of 20 (25%) iRBD patients presenting normal VEMPs, compared with 17/22 (77%) controls with normal battery (*p* = 0.002) (Figure 3).

The features of VEMPs in iRBD and controls are displayed in Table 2. In comparison with controls, there was a significant decrease in the amplitude of all VEMPs in iRBD subjects (*p* < 0.0001 for cVEMP and oVEMP and *p* = 0.001 for mVEMP). The peak latency of the first wave of the mVEMP and oVEMP, as well as the onset latency of the mVEMP, were significantly delayed in iRBD patients compared with controls. The VEMP score was significantly higher in iRBD patients both for each single VEMP (cVEMP: *p* = 0.02; mVEMP and oVEMP: *p* = 0.01) and for the total VEMP score (iRBD patients: 4.7 ± 4.5; controls: 0.5 ± 1.34, *p* < 0.0001).

**Table 2:** Comparison of morphological characteristics of the cVEMP, mVEMP and oVEMP between iRBD patients and controls.

**VEMP**	**Parameter**	**iRBD**	**Controls**	***p***
cVEMP	p13 Onset (ms)	8.95 ± 1.49	8.7 ± 1.62	n.s.
	p13 peak latency (ms)	12.78 ± 1.22	12.62 ± 1.33	n.s.
	n23 peak latency (ms)	19.47 ± 2.5	19.75 ± 1.64	n.s.
	p13/n23 amplitude	0.66 ± 0.31	1.01 ± 0.36	<0.0001
	Mean EMG (μV)	92.38 ± 41.62	110.6 ± 65.83	n.s.
	Interpeak difference (ms)	6.7 ± 2.2	7.83 ± 1.28	0.03
	Amplitude asymmetry ratio	24.08 ± 20.62	14.43 ± 11.34	n.s.
	cVEMP score	1.60 ± 1.03	0.09 ± 0.43	0.02
mVEMP	p11 Onset (ms)	10.05 ± 0.92	9.18 ± 1.21	<0.0001
	p11 peak latency (ms)	12.84 ± 0.99	11.94 ± 1.01	<0.0001
	p11 amplitude	0.34 ± 0.23	0.4 ± 0.15	0.001
	Mean EMG (μV)	75.96 ± 33.43	69.36 ± 30.65	n.s.
	Interpeak difference (ms)	8.46 ± 2.13	8.11 ± 1.81	n.s.
	Amplitude asymmetry ratio	16.09 ± 9.84	16.1 ± 15.81	n.s.
	mVEMP score	3.60 ± 4.02	0.73 ± 2.59	0.01
oVEMP	n10 Onset (ms)	7.59 ± 1.24	7.43 ± 0.94	n.s.
	n10 peak latency (ms)	9.5 ± 1.25	9.0 ± 1.06	0.02
	n15 peak latency (ms)	13.36 ± 1.67	13.27 ± 1.49	n.s.
	n10/p15 amplitude	0.49 ± 0.3	1.12 ± 0.37	<0.0001
	Mean EMG (μV)	11.31 ± 5.02	9.66 ± 3.69	n.s.
	Interpeak difference (ms)	4.01 ± 1.16	4.27 ± 1.14	n.s.
	Amplitude asymmetry ratio	28.94 ± 17.83	25.11 ± 16.6	n.s.
	oVEMP score	2.15 ± 2.58	0.46 ± 0.21	0.01

*Amplitude is expressed as ratio between the peak or peak-to peak amplitude (in μV) and background EMG activity (in μV). Statistics: Mann Whitney U-test. n.s, non-significant*.

**Figure 4:**
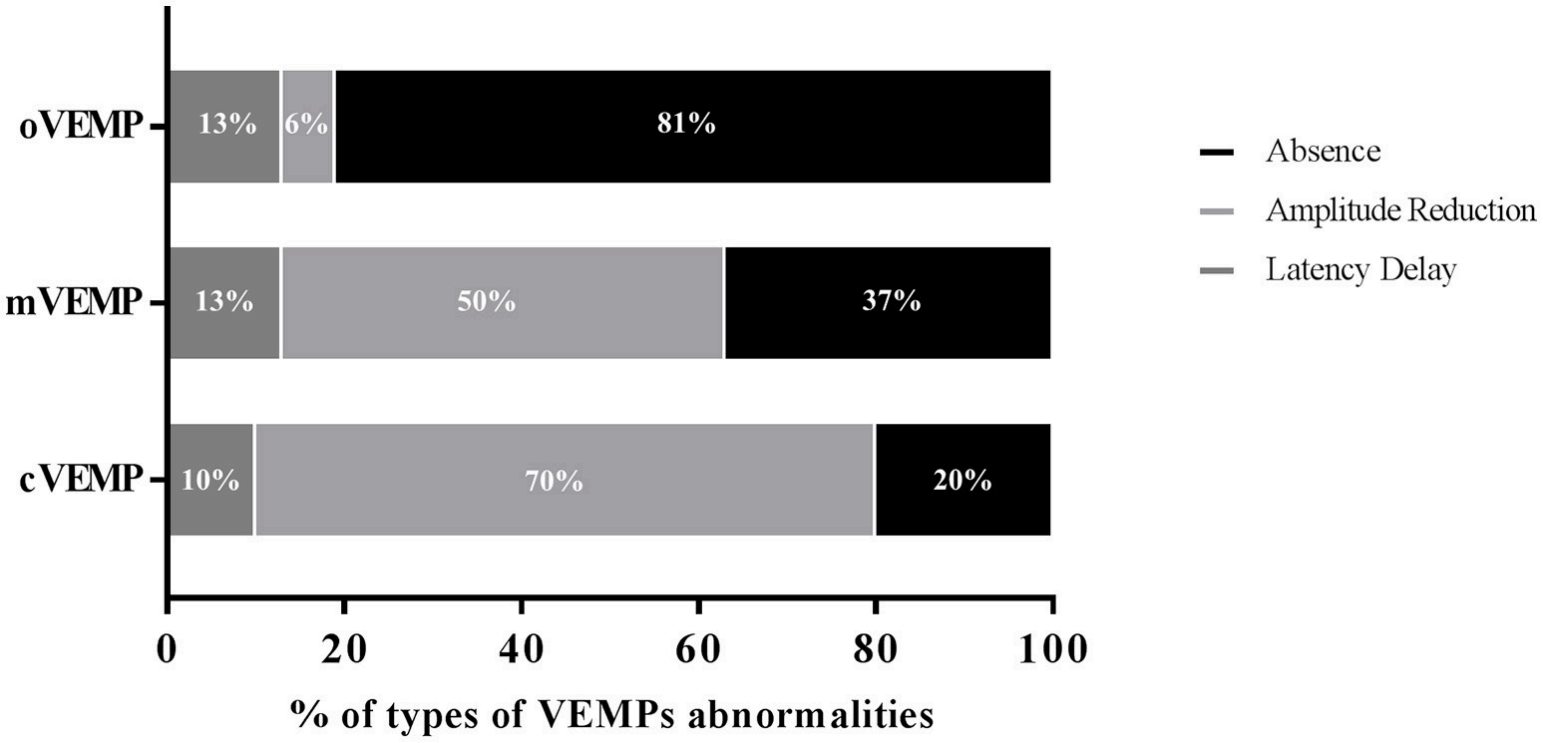
Pattern of the abnormalities detected in iRBD patients in each of the three VEMPs. cVEMP, cervical VEMP; mVEMP, masseter VEMP; oVEMP, ocular VEMP.

With regard to the side of VEMP abnormality, 8 iRBD patients showed monolateral cVEMP alterations while the deficit was bilateral in only one patient. The oVEMP appeared altered monolaterally in 4 iRBD subjects and bilaterally in 6 patients. The mVEMP alterations were present bilaterally following both unilateral and bilateral stimulation in 7 iRBD patients and monolaterally in 6. When considering the pattern of the abnormalities detected (Figure 4), absence was mostly frequent in the oVEMP (81.2%) and amplitude reduction was most represented in the cVEMP (70%) and in the mVEMP (50%). By contrast, latency delay was the least represented alteration and did not differ in frequency among the three VEMPs.

Finally, analysis of correlations between neurophysiological findings and clinical and demographic characteristics of iRBD patients, was performed: higher oVEMP scores correlated significantly with lower scores on BBS (ρ: −0.509, *p* = 0.02). No other correlations were found, particularly, no correlation between severity of VEMPs alterations as assessed with VEMPs cores and disease duration was detected.

## Discussion

To the best of our knowledge, this is the first study in which a thorough neurophysiological test battery has been employed to assess brainstem physiology alterations in patients with iRBD. The combined use of cVEMP, mVEMP and oVEMP allows a non-specific indirect measure of assessment of brainstem function along its whole length that, in the study of neurological diseases, has the potential of disclosing alterations in regions which may otherwise be undetectable at clinical and radiological evaluation (7, 8). This characteristic makes VEMPs potentially suited for studying the brainstem in neurological conditions even at the very early stages of diseases, that is, when overt symptomatic or structural impairments have not ensued yet. Previous works performed on patients with PD had shown that VEMPs were all consistently altered since the very early motor stages of disease; interestingly, these studies also showed that VEMP abnormalities were highly correlated with the presence of RBD symptoms as assessed by the RBD-SQ scale (5, 6).

Healthy subjects with normal hearing are not supposed to have alterations of any of the VEMPs. Despite this, in our control sample, there have been some cases of VEMP abnormalities. Some studies have previously demonstrated that the morphology of VEMPs can be influenced by age and that AC-click-evoked VEMPs morphology over the 55–65 age group can be significantly affected (23–25). This would be the case also in our population of controls, that has a mean age of 66.5 years. A common cause of VEMPs alteration is a suboptimal hearing capacity, which can occur frequently in elderly. However, all participants in this study tested negative to a preliminary audiometric examination; moreover, this possibility can be ruled out by also taking into consideration that none of the controls had more than one altered VEMP. Therefore, we think that the significant difference that we have found between iRBD patients and healthy controls can be ascribable to underlying pathological phenomena in the brainstem which are taking place in iRBD.

RBD, as expression of a preclinical phase of neurodegenerative diseases such as synucleinopathies, is thought to arise from a multifactorial cellular derangement occurring very early from motor onset of disease (26) according to a proposed stereotyped pathological process (4). Our RBD cohort was free of any sign of motor impairment, as assessed by the UPDRS score part III. However, up to 45% of patients showed, in concomitance, the presence of a symptom that has been linked to a higher PD risk, pointing out to the possibility that a subtle neuronal degeneration is taking place. Unfortunately, no risk factor able to predict prospectively the conversion from RBD to a neurodegenerative disease can be indicated (27), the only tool that has demonstrated reliable predictive value being ^123^I-FP-DAT-SPECT (28). In this light, we believe that the consistent finding of VEMPs dysfunction in our cohort is potentially relevant in the context of the search of biomarkers for neurodegeneration in patients displaying prodromal conditions. In order to confirm this hypothesis, a longitudinal study of this cohort is warranted. This latter hypothesis is reasonably corroborated by previous neurophysiological data obtained in PD, which is a neurodegenerative disease that is associated with high frequency with RBD as its first symptom (29). Previous works have shown that the VEMP battery used in the present study was able to disclose a significant difference in brainstem functional integrity between PD patients and age-matched healthy controls, with significant neurophysiological-clinical correlation with the presence of symptoms suggestive of RBD, as measured with the RBD-Screening Questionnaire (5, 6). These data suggest that this neurophysiological investigation may have potential in the temporal observation of selected cohorts of people considered at risk for neurodegenerative conditions, such as PD. With regard to brainstem pathophysiology, several regions using different neurotransmitters have been proposed as critical for the generation of REM sleep (2). Dysfunction of the noradrenergic locus coeruleus/subcoeruleus complex, located in the posterior area of the rostral pons, has recently gained attention as a key factor in the generation of REM sleep without atonia, a hallmark of RBD (30). Moreover, acetylcholine-mediated inputs from the pedunculopontine and laterodorsal tegmentum are believed to modulate REM sleep (31). Finally, GABAergic neurons located in the dorsal paragigantocellular nucleus, modulate the activity of nearby nuclei such as the dorsal raphe, therefore facilitating the entrance to REM sleep phase (32). It is conceivable that, in RBD, due to a derangement of these critical regions for the homeostasis of REM sleep, the central reflex volleys of VEMPs might be affected by dysfunctional interneuronal loops mediated by one or more neurotransmitters involved also in the pathophysiology of this condition. This hypothesis is supported by some findings: the medial vestibular nuclei project diffusely to brainstem and hypothalamic areas implicated in REM sleep (33); a noradrenergic connection between the locus coeruleus and the vestibular nuclei exists (34) and it has been proved that vestibular nuclei activity is modulated by noradrenergic inputs (35, 36). Additionally, connections between the dorsal raphe and the vestibular nuclei have been demonstrated in the animal model (37). In further support, Diffusion Tensor Imaging studies have demonstrated an alteration of axial diffusivity (a marker of axonal damage) in the brainstem of RBD patients (38). This latter finding may also explain the high rates of representation of absence and amplitude reduction in iRBD for all VEMPs considered.

Although in our iRBD cohort, the single VEMP scores were not significantly different, a tendency for a caudo-rostral gradient of severity of abnormalities was observed, in that alterations on more rostral VEMPs, such as the mVEMP and particularly the oVEMP, tended to be more severe compared to cVEMP. For example, there was a tendency for oVEMP alterations to be bilaterally represented more frequently than in the mVEMP and cVEMP, which are integrated at a more caudal level. Furthermore, the rate of absences and low amplitudes encountered in cVEMP, was significantly lower than what found both in mVEMP and oVEMP.

The central pathway of the cVEMP reflex volley is represented by the vestibulospinal tract that runs away from the brainstem to the XI cranial nerve nucleus in the upper segments of the spinal cord (12). The mVEMP is likely mediated by a bilateral and crossed pathway that links the medial vestibular nucleus and the prepositus hypoglossi nucleus in the medulla to the motor trigeminal nucleus in the pons (39, 40). The oVEMP reflex circuit involves an ascending crossed pathway from the VIII to the contralateral III cranial nerve nuclei (41), thus running across the whole pons and part of the mesencephalon, with higher chance to meet cellular connections belonging to the reticular formation. The differences in the central circuits traveled by the three VEMPs may account for the small but notable diversity encountered in the rate and typology of alterations of each of them. A potentially important region in this sense is represented by the pedunculo-pontine nucleus (PPN) that, besides its involvement in the generation and modulation of REM sleep (31), plays a role in the stabilization of posture and its maintenance during locomotion (42). Furthermore, there are evidences about cholinergic connections between vestibular nuclei and more rostral areas in the reticular formation (43), particularly the PPN (44). This might explain the consistent correlation between the oVEMP score and low scores on postural instability, assessed by the BBS, that has been found in our iRBD cohort. None of our patients ever complained of falls and presence of known causes of postural instability was indeed an exclusion criterion. The presence of postural instability in RBD is a matter of debate: although, in full blown PD, this symptom might not seem to associate with RBD condition (45), patients with iRBD show increased postural sway, associated with alteration of stereopsis, compared with controls (46). These data suggest that dysfunctions affecting vestibulo-mesencephalic connections may be present at subclinical level in iRBD but further studies are needed to test this hypothesis.

In conclusion, this study evidences consistent signs of brainstem dysfunction detected through a battery of VEMPs in a group of iRBD patients without any motor sign suggestive of a neurodegenerative disease. This finding adds knowledge about the ongoing brainstem degeneration during this condition that, in a high percentage of cases, ultimately leads to the development of a synucleinopathy. Further studies are warranted to correlate the presence of abnormal VEMPs in iRBD with that of other prodromal markers for future α-synucleinopathy onset. Furthermore, a follow-up is suggested to determine whether those patients with evidence of VEMPs alterations may be at higher risk of future conversion to either PD, Multiple System Atrophy or Dementia with Lewy Bodies.

## Author contributions

FD and MP conception and design of the study. EdN, FG, IL, BM, AM and MF acquisition and analysis of data. EdN, FD, MP, and FG drafting and reviewing of the manuscript.

### Conflict of interest statement

The authors declare that the research was conducted in the absence of any commercial or financial relationships that could be construed as a potential conflict of interest.

## References

[1] Schenck CH Bundlie SR Ettinger MG Mahowald MW. Chronic behavioral disorders of human REM sleep: a new category of parasomnia. Sleep (1986) 9:293–308. 10.1093/sleep/9.2.293 3505730

[2] Fraigne JJ Torontali ZA Snow MB Peever JH. REM Sleep at its core - circuits, neurotransmitters, and pathophysiology. Front Neurol. (2015) 6:123. 10.3389/fneur.2015.00123 26074874 PMC4448509

[3] Postuma RB Gagnon JF Vendette M Montplaisir JY. Idiopathic REM sleep behavior disorder in the transition to degenerative disease. Mov Disord. (2009) 24:2225–32. 10.1002/mds.22757 19768814

[4] Braak H Del Tredici K Rub U De Vos RA Jansen Steur EN Braak E. Staging of brain pathology related to sporadic Parkinson's disease. Neurobiol Aging (2003) 24:197–211. 10.1016/S0197-4580(02)00065-9 12498954

[5] De Natale ER Ginatempo F Paulus KS Manca A Mercante B Pes GM. Paired neurophysiological and clinical study of the brainstem at different stages of Parkinson's Disease. Clin Neurophysiol. (2015) 126:1871–8. 10.1016/j.clinph.2014.12.017 25622530

[6] De Natale ER Ginatempo F Paulus KS Pes GM Manca A Tolu E. Abnormalities of vestibular-evoked myogenic potentials in idiopathic Parkinson's disease are associated with clinical evidence of brainstem involvement. Neurol Sci. (2015) 36:995–1001. 10.1007/s10072-014-2054-4 25567081

[7] Magnano I Pes GM Pilurzi G Cabboi MP Ginatempo F Giaconi E. Exploring brainstem function in multiple sclerosis by combining brainstem reflexes, evoked potentials, clinical and MRI investigations. Clin Neurophysiol. (2014) 125:2286–96. 10.1016/j.clinph.2014.03.016 24745338

[8] Magnano I Pes GM Cabboi MP Pilurzi G Ginatempo F Achene A. Comparison of brainstem reflex recordings and evoked potentials with clinical and MRI data to assess brainstem dysfunction in multiple sclerosis: a short-term follow-up. Neurol Sci. (2016) 37:1457–65. 10.1007/s10072-016-2604-z 27177651

[9] Peter A Hansen ML Merkl A Voigtlander S Bajbouj M Danker-Hopfe H. REM sleep behavior disorder and excessive startle reaction to visual stimuli in a patient with pontine lesions. Sleep Med. (2008) 9:697–700. 10.1016/j.sleep.2007.10.009 18060836

[10] Zoetmulder M Biernat HB Nikolic M Korbo L Jennum PJ. Sensorimotor gating deficits in multiple system atrophy: comparison with Parkinson's disease and idiopathic REM sleep behavior disorder. Parkinsonism Relat Disord. (2014) 20:297–302. 10.1016/j.parkreldis.2013.11.018 24355363

[11] Berg K Wood-Dauphine S Williams J Gayton D Measuring balance in the elderly: preliminary development of an instrument. Physiother Can. (1989) 41:304–11. 10.3138/ptc.41.6.304

[12] Colebatch JG Halmagyi GM Skuse NF. Myogenic potentials generated by a click-evoked vestibulocollic reflex. J Neurol Neurosurg Psychiatr. (1994) 57:190–7. 10.1136/jnnp.57.2.190 8126503 PMC1072448

[13] Deriu F Tolu E Rothwell JC. A short latency vestibulomasseteric reflex evoked by electrical stimulation over the mastoid in healthy humans. J Physiol. (2003) 553:267–79. 10.1113/jphysiol.2003.047274 12949229 PMC2343496

[14] Deriu F Tolu E Rothwell JC. A sound-evoked vestibulomasseteric reflex in healthy humans. J Neurophysiol. (2005) 93:2739–51. 10.1152/jn.01005.2004 15601734

[15] Rosengren SM Mcangus Todd NP Colebatch JG. Vestibular-evoked extraocular potentials produced by stimulation with bone-conducted sound. Clin Neurophysiol. (2005) 116:1938–48. 10.1016/j.clinph.2005.03.019 15979939

[16] Deriu F Ortu E Capobianco S Giaconi E Melis F Aiello E. Origin of sound-evoked EMG responses in human masseter muscles. J Physiol. (2007) 580:195–209. 10.1113/jphysiol.2006.123240 17234698 PMC2075422

[17] Deriu F Giaconi E Rothwell JC Tolu E. Reflex responses of masseter muscles to sound. Clin Neurophysiol. (2010) 121:1690–9. 10.1016/j.clinph.2009.11.093 20447862

[18] Papathanasiou ES Murofushi T Akin FW Colebatch JG. International guidelines for the clinical application of cervical vestibular evoked myogenic potentials: an expert consensus report. Clin Neurophysiol. (2014) 125:658–66. 10.1016/j.clinph.2013.11.042 24513390

[19] Rosengren SM Welgampola MS Colebatch JG. Vestibular evoked myogenic potentials: past, present and future. Clin Neurophysiol. (2010) 121:636–51. 10.1016/j.clinph.2009.10.016 20080441

[20] Fukutake T Kuwabara S Kaneko M Kojima S Hattori T. Sensory impairments in spinal multiple sclerosis: a combined clinical, magnetic resonance imaging and somatosensory evoked potential study. Clin Neurol Neurosurg. (1998) 100:199–204. 10.1016/S0303-8467(98)00045-6 9822842

[21] Leocani L Rovaris M Boneschi FM Medaglini S Rossi P Martinelli V. Multimodal evoked potentials to assess the evolution of multiple sclerosis: a longitudinal study. J Neurol Neurosurg Psychiatr. (2006) 77:1030–5. 10.1136/jnnp.2005.086280 16735397 PMC2077734

[22] Gabelic T Krbot Skoric M Adamec I Barun B Zadro I Habek M. The vestibular evoked myogenic potentials (VEMP) score: a promising tool for evaluation of brainstem involvement in multiple sclerosis. Eur J Neurol. (2015) 22:261–9, e21. 10.1111/ene.12557 25196120

[23] Welgampola MS Colebatch JG. Vestibulocollic reflexes: normal values and the effect of age. Clin Neurophysiol. (2001) 112:1971–9. 10.1016/S1388-2457(01)00645-9 11682335

[24] Govender S Rosengren SM Todd NP Colebatch JG. Ocular vestibular evoked myogenic potentials produced by impulsive lateral acceleration in unilateral vestibular dysfunction. Clin Neurophysiol. (2011) 122:2498–504. 10.1016/j.clinph.2011.04.024 21640646

[25] Rosengren SM Govender S Colebatch JG. Ocular and cervical vestibular evoked myogenic potentials produced by air- and bone-conducted stimuli: comparative properties and effects of age. Clin Neurophysiol. (2011) 122:2282–9. 10.1016/j.clinph.2011.04.001 21550301

[26] Grinberg LT Rueb U Alho AT Heinsen H. Brainstem pathology and non-motor symptoms in PD. J Neurol Sci. (2010) 289:81–8. 10.1016/j.jns.2009.08.021 19758601

[27] Postuma RB Iranzo A Hogl B Arnulf I Ferini-Strambi L Manni R. Risk factors for neurodegeneration in idiopathic rapid eye movement sleep behavior disorder: a multicenter study. Ann Neurol. (2015) 77:830–9. 10.1002/ana.24385 25767079 PMC5769479

[28] Iranzo A Santamaria J Valldeoriola F Serradell M Salamero M Gaig C. Dopamine transporter imaging deficit predicts early transition to synucleinopathy in idiopathic rapid eye movement sleep behavior disorder. Ann Neurol. (2017) 82:419–28. 10.1002/ana.25026 28833467

[29] Mahowald MW Schenck CH The “when” and “where” of alpha-synucleinopathies: Insights from REM sleep behavior disorder. Neurology (2018) 91:435–6. 10.1212/WNL.0000000000006129 30089622

[30] Garcia-Lorenzo D Longo-Dos Santos C Ewenczyk C Leu-Semenescu S Gallea C Quattrocchi G. The coeruleus/subcoeruleus complex in rapid eye movement sleep behaviour disorders in Parkinson's disease. Brain (2013) 136:2120–9. 10.1093/brain/awt152 23801736 PMC3692035

[31] Van Dort CJ Zachs DP Kenny JD Zheng S Goldblum RR Gelwan NA. Optogenetic activation of cholinergic neurons in the PPT or LDT induces REM sleep. Proc Natl Acad Sci USA. (2015) 112:584–9. 10.1073/pnas.1423136112 25548191 PMC4299243

[32] Luppi PH Gervasoni D Verret L Goutagny R Peyron C Salvert D. Paradoxical (REM) sleep genesis: the switch from an aminergic-cholinergic to a GABAergic-glutamatergic hypothesis. J Physiol Paris (2006) 100:271–83. 10.1016/j.jphysparis.2007.05.006 17689057

[33] Horowitz SS Blanchard J Morin LP. Medial vestibular connections with the hypocretin (orexin) system. J Comp Neurol. (2005) 487:127–46. 10.1002/cne.20521 15880498

[34] Schuerger RJ Balaban CD. Organization of the coeruleo-vestibular pathway in rats, rabbits, and monkeys. Brain Res Brain Res Rev. (1999) 30:189–217. 10.1016/S0165-0173(99)00015-6 10525175

[35] Di Mauro M Bronzi D Li Volsi G Licata F Lombardo P Santangelo F. Noradrenaline modulates neuronal responses to GABA in vestibular nuclei. Neuroscience (2008) 153:1320–31. 10.1016/j.neuroscience.2008.02.014 18440712

[36] Barresi M Caldera M Grasso C Li Volsi G Licata F Santangelo F. Noradrenergic modulation of neuronal responses to glutamate in the vestibular complex. Neurosci Lett. (2009) 464:173–8. 10.1016/j.neulet.2009.08.035 19699262

[37] Halberstadt AL Balaban CD. Selective anterograde tracing of the individual serotonergic and nonserotonergic components of the dorsal raphe nucleus projection to the vestibular nuclei. Neuroscience (2007) 147:207–23. 10.1016/j.neuroscience.2007.03.049 17507165 PMC2093990

[38] Unger MM Belke M Menzler K Heverhagen JT Keil B Stiasny-Kolster K. Diffusion tensor imaging in idiopathic REM sleep behavior disorder reveals microstructural changes in the brainstem, substantia nigra, olfactory region, and other brain regions. Sleep (2010) 33:767–73. 10.1093/sleep/33.6.767 20550017 PMC2881532

[39] Giaconi E Deriu F Tolu E Cuccurazzu B Yates BJ Billig I. Transneuronal tracing of vestibulo-trigeminal pathways innervating the masseter muscle in the rat. Exp Brain Res. (2006) 171:330–9. 10.1007/s00221-005-0275-8 16307240 PMC2396390

[40] Cuccurazzu B Deriu F Tolu E Yates BJ Billig I. A monosynaptic pathway links the vestibular nuclei and masseter muscle motoneurons in rats. Exp Brain Res. (2007) 176:665–71. 10.1007/s00221-006-0834-7 17216144 PMC2684793

[41] Weber KP Rosengren SM Michels R Sturm V Straumann D Landau K. Single motor unit activity in human extraocular muscles during the vestibulo-ocular reflex. J Physiol. (2012) 590:3091–101. 10.1113/jphysiol.2011.226225 22526888 PMC3406392

[42] Benarroch EE. Pedunculopontine nucleus: functional organization and clinical implications. Neurology (2013) 80:1148–55. 10.1212/WNL.0b013e3182886a76 23509047

[43] Smith P Darlington C. Pharmacology of the vestibular system. Bailliere's Clin Neurol. (1994) 3:467–84. 7874403

[44] Woolf NJ Butcher LL. Cholinergic systems in the rat brain: IV. Descending projections of the pontomesencephalic tegmentum. Brain Res Bull. (1989) 23:519–40. 10.1016/0361-9230(89)90197-4 2611694

[45] Benninger DH Michel J Waldvogel D Candia V Poryazova R Van Hedel HJ REM sleep behavior disorder is not linked to postural instability and gait dysfunction in Parkinson. Mov Disord. (2010) 25:1597–604. 10.1002/mds.23121 20629146

[46] Chen TZ Xu GJ Zhou GA Wang JR Chan P Du YF. Postural sway in idiopathic rapid eye movement sleep behavior disorder: a potential marker of prodromal Parkinson's disease. Brain Res. (2014) 1559:26–32. 10.1016/j.brainres.2014.02.040 24602694

